# Design and evaluation of an intelligent reduction robot system for the minimally invasive reduction in pelvic fractures

**DOI:** 10.1186/s13018-022-03089-2

**Published:** 2022-04-04

**Authors:** Chunpeng Zhao, Yu Wang, Xinbao Wu, Gang Zhu, Shuchang Shi

**Affiliations:** 1 Department of Orthopedics and Traumatology, Beijing Jishuitan Hospital, Beijing, 100035 China; 2 School of Biological Science and Medical Engineering, Beihang University, Beijing, 100083 China; 3 Beijing Advanced Innovation Center for Biomedical Engineering, Beihang University, Beijing, 100083 China; 4 Rossum Robot Co., Ltd., Beijing, 100083 China

**Keywords:** Pelvic fracture, Reduction, Robot-assisted fracture reduction, Registration

## Abstract

**Introduction:**

Pelvic fracture is a severe high-energy injury with the highest disability and mortality of all fractures. Traditional open surgery is associated with extensive soft tissue damages and many complications. Minimally invasive surgery potentially mitigates the risks of open surgical procedures and is becoming a new standard for pelvic fracture treatment. The accurate reduction has been recognized as the cornerstone of minimally invasive surgery for pelvic fracture. At present, the closed reduction in pelvic fractures is limited by the current sub-optimal 2D intra-operative imaging (fluoroscopy) and by the high forces of soft tissue involved in the fragment manipulation, which might result in fracture malreduction. To overcome these shortcomings and facilitate pelvic fracture reduction, we developed an intelligent robot-assisted fracture reduction (RAFR) system for pelvic fracture.

**Methods:**

The presented method is divided into three parts. The first part is the preparation of 20 pelvic fracture models. In the second part, we offer an automatic reduction algorithm of our robotic reduction system, including Intraoperative real-time 3D navigation, reduction path planning, control and fixation, and robotic-assisted fracture reduction. In the third part, image registration accuracy and fracture reduction accuracy were calculated and analyzed.

**Results:**

All 20 pelvic fracture bone models were reduced by the RAFR system; the mean registration error E1 of the 20 models was 1.29 ± 0.57 mm. The mean reduction error E2 of the 20 models was 2.72 ± 0.82 mm. The global error analysis of registration and reduction results showed that higher errors are mainly located at the edge of the pelvis, such as the iliac wing.

**Conclusion:**

The accuracy of image registration error and fracture reduction error in our study was excellent, which could reach the requirements of the clinical environment. Our study demonstrated the precision and effectiveness of our RAFR system and its applicability and usability in clinical practice, thus paving the way toward robot minimally invasive pelvic fracture surgeries.

## Introduction

Pelvic fractures account for 2–8% of all fractures. It is a severe high-energy injury with a disability rate of 60% and a mortality rate of more than 13% [1, 2]. The main characteristics of pelvic fractures are pelvic ring fracture and fracture displacement. Accurate reduction for unstable pelvic fractures has been recognized as the cornerstone of pelvic fracture treatment [3, 4]. It has been suggested that improved reduction correlates with better functional outcome [5–8] and that anatomic reduction in the pelvic ring is as important as simple stabilization [9]

The early concept of pelvic fracture treatment often requires an open surgical procedure that provides accurate reduction but relies extensively on the surgeon’s expertise. The exposure of the fractured location is also associated with extensive soft tissue damages, a higher risk of infection, more extended hospitalization and rehabilitation time, and higher costs [10]. With the advancement of medical technology and the popularization of minimally invasive concepts, closed reduction techniques allow the doctor to manipulate the fracture fragments through small incisions fleshly, thereby potentially mitigating the risks of open surgical procedures. At the same time, it is emphasized that the closed reduction technique also has certain advantages in the second-stage anatomical reconstruction surgery and is becoming a new standard for fracture treatment [11]. However, closed reduction techniques are limited by static two-dimensional (2D) intra-operative fluoroscopic imaging often inadequate for three-dimensional (3D) fragment alignments, necessitating multiple intra-operative images, which leads to prolonged radiation exposure of the patient and medical staff [12]. During the reduction process, the high forces between muscular attachments and bone fracture fragments often prevent correct reduction movements and occasionally result in fracture malreduction. Moreover, the manual reduction technique is a trial-and-error process, which cannot guarantee a precise reduction. These problems are particularly evident when dealing with pelvic fractures where accurately anatomical reduction is a 3D problem, typically difficult to resolve using 2D imaging.

Various computer- and robot-assisted fracture reduction systems were developed to overcome these shortcomings and facilitate fracture reduction. However, most robot-assisted fracture reduction systems established previously were designed for lower extremity diaphyseal fracture. Because of the complicated and critical trauma caused by pelvic fracture, more critical issues (e.g., reduction path and force control) need to be considered. Studies on robot-assisted pelvic fracture reduction were still in the preliminary stage. After careful investigation, our team developed an intelligent robot-assisted fracture reduction (RAFR) system for pelvic fracture, utilizing modern techniques such as three-dimensional (3D) imaging data, navigation, and robotics. Our research activities aim to introduce an intelligent RAFR system for pelvic fracture, which concentrates on proposals for optimized reduction pathway planning to achieve smooth reductions and simulates the clinical operation. The RAFR system is described in the method of this paper. We completed the automatic reduction of 20 pelvic fracture 3D printed bone models with intelligent RAFR and presented some results based on the simulation. Then, the accuracy of image registration and fracture reduction was calculated and analyzed. It is important to note that we only work with the reduction in pelvic fractures. An extension to screw placement is the task of further work.

## Method

The presented method is divided into three parts. The first part is the preparation of 20 pelvic fracture models. In the second part, we offer an automatic reduction algorithm of our robotic reduction system, including Intraoperative real-time 3D navigation, reduction path planning, control and fixation, and robotic-assisted fracture reduction. In the third part, image registration accuracy and fracture reduction accuracy were calculated and analyzed.

All experiments were performed in the laboratory. We obtained approval for the use of patient CT from our institution's ethics committee. This article does not contain patient data. Human subjects were recruited to perform computer‐based tasks only. The subjects were informed about the objectives and the study format and were only recruited if they agreed to participate in the study.

Our study was approved by the Ethic Committee of Beijing Jishuitan Hospital (No: 202006-13). This research is supported by the National Natural Science Foundation of China (NSFC61871019), Beijing science and technology project (Z201100005420033), and Natural Science Foundation of Beijing (19L2011).

The metrics chosen for the RAFR system evaluation were (1) the registration accuracy expressed as the mean registration error measured after the registration; (2) the fracture reduction accuracy expressed as the mean reduction error measured after the physical reductions. (3) The mean registration error E1 and reduction error E2 in different anatomy landmark points. (4)The registration error distribution of the global 3D point cloud on the fracture side of one model. (5) The reduction error distribution of the global 3D point cloud on the fracture side of one model.

### Pelvic fracture models

The data come from normal pelvic CT scans of 20 anonymous patients (twelve males and eight females, age ranging from 25 to 72 years) from Beijing Jishuitan Hospital. All of the above patients underwent abdominal CT scans due to other diseases and had no pelvic injury. The normal pelvic CT data of these 20 patients were imported in digital imaging and communication in medicine (DICOM) format into Mimics software (Materialise, Haasrode, Belgium) to create 20 pelvic 3D models. Then, 20 pelvic synthetic 3D printing models were created by a 3D printer (RS6000, union tech co. Ltd, China).

**Fig. 1:**
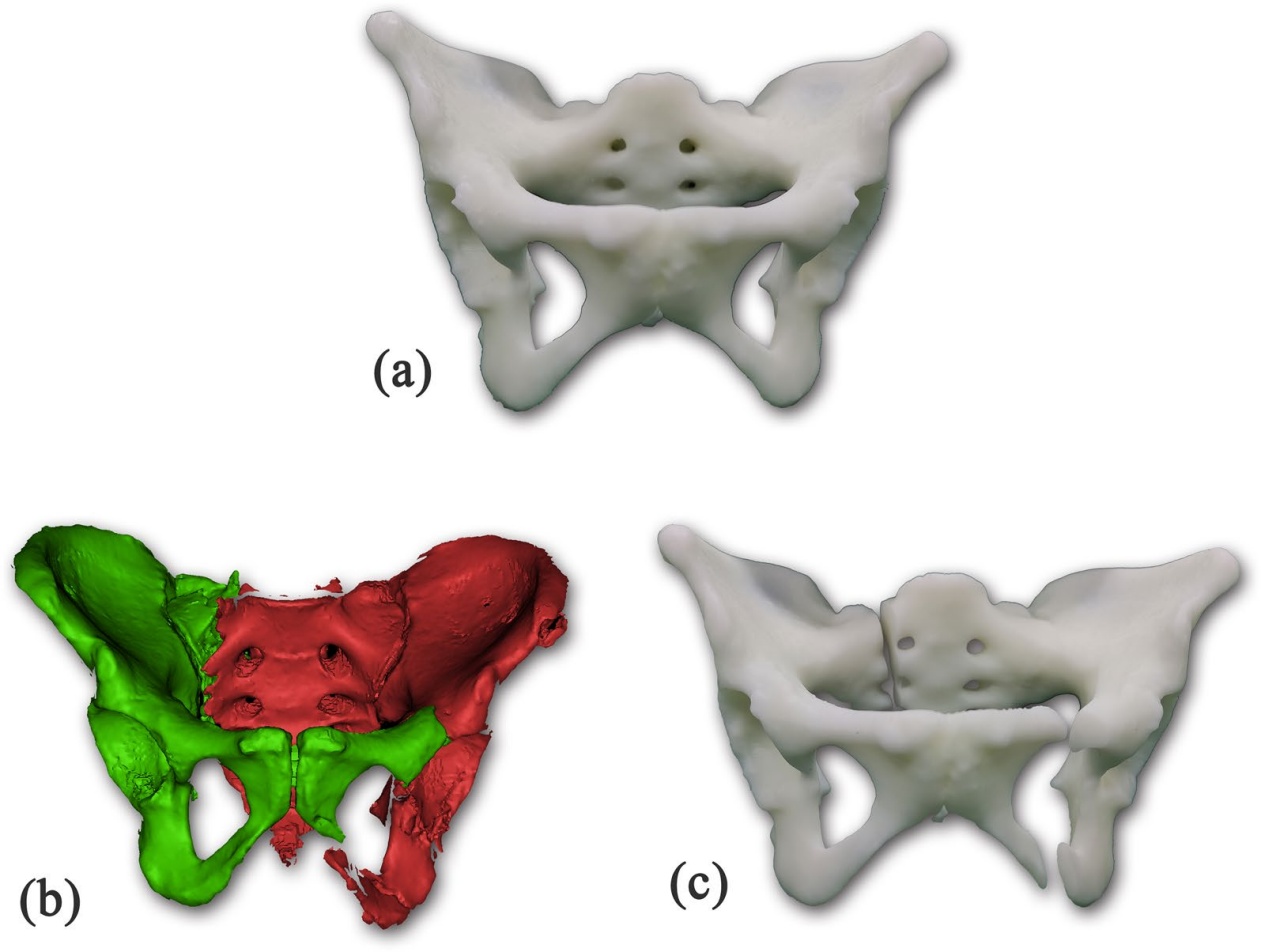
Model making for the experiment **a** 3D printing of the complete pelvis of healthy patients. **b** Fracture morphology reconstructed by CT scan of patients with pelvic fracture. **c** Fracture pelvis model made by osteotomy according to the fracture morphology

We selected 20 clinical cases of pelvic fracture in our hospital, which was classified into 6 cases of type B1, 8 cases of type B2, and 6 cases of type C1 according to the Tile classification [13]. Based on the fracture morphology of these 20 clinical pelvic fracture cases, we performed osteotomy on 20 pelvic 3D printing models to simulate these 20 clinical cases of pelvic fractures. At last, we created 20 pelvic fracture models for our study (Fig. 1).

**Fig. 2:**
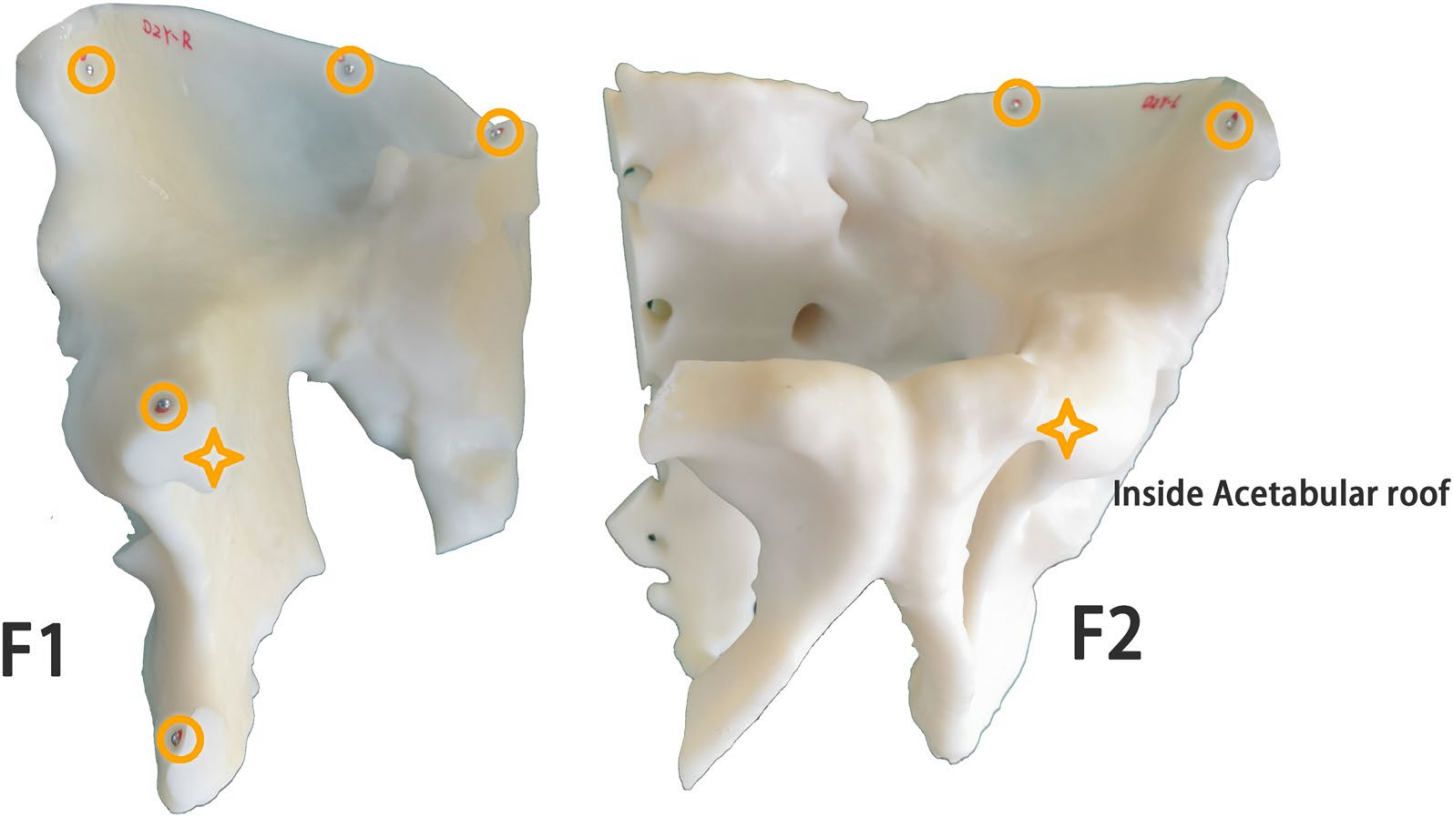
The position of the metal ball used for accuracy analysis

We have selected the contralateral healthy hemipelvis as F1 and the fractured hemipelvis as F2 for each pelvic model, as shown in Fig. 2. The 3 mm diameter metal balls were pasted on the pelvis model that the position may be of interest to the doctor for accuracy analysis. We pasted six metal balls on the fractured side. The positions of the six balls were: the anterior iliac wing, the posterior iliac wing, the acetabulum, the sacrum, the suprapubic branch, and the inferior pubic branch. Three metal balls were also pasted on the healthy side, located on the anterior iliac wing, the posterior iliac wing, and the lateral side of the acetabulum.

### Algorithm of our RAFR system

**Fig. 3:**
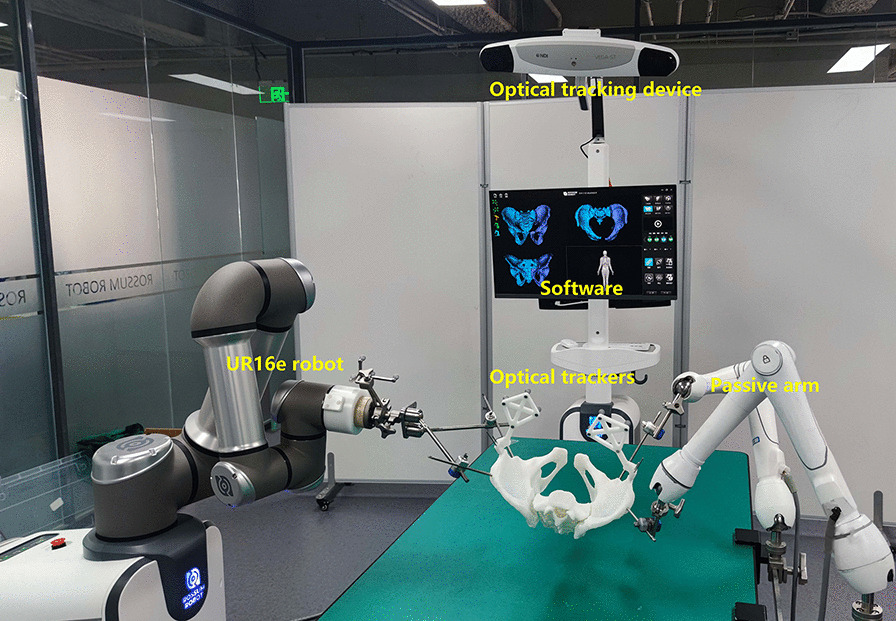
Intelligent robot-assisted fracture reduction system for pelvic fracture

Our RAFR system (Fig. 3) consists of four main parts: pelvic fracture reduction software (including reduction path planning software, intraoperative navigation, and registration software), photoelectric tracking device (NDI Polaris Vega and trackers), pelvic holding equipment, and reduction robot (UR16e). The optical tracking device is connected to the pelvis model and the robot for real-time tracking during the reduction. The holding equipment is connected to the operating table through a designed U-shaped device. The healthy side holding equipment consists of two six-degree-of-freedom (DOF) electronically controlled passive arms to achieve stable holding of the healthy side pelvis.

**Fig. 4:**
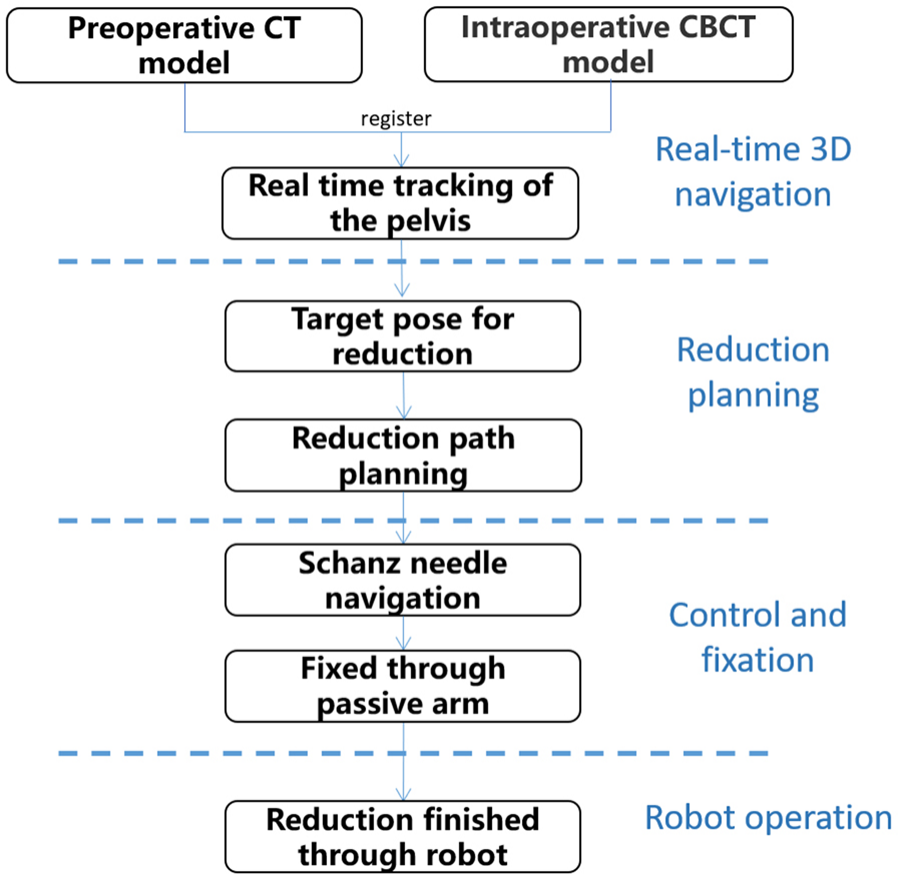
Algorithm of Intelligent robot-assisted fracture reduction system for pelvic fracture

There are four steps of our RAFR system, including intraoperative real-time 3D navigation, reduction planning, intraoperative holding and fixation, and robot-assisted reduction. Figure 4 shows the algorithm of our RAFR system for pelvic fracture reduction.

#### Intraoperative real-time 3D navigation

**Fig. 5:**
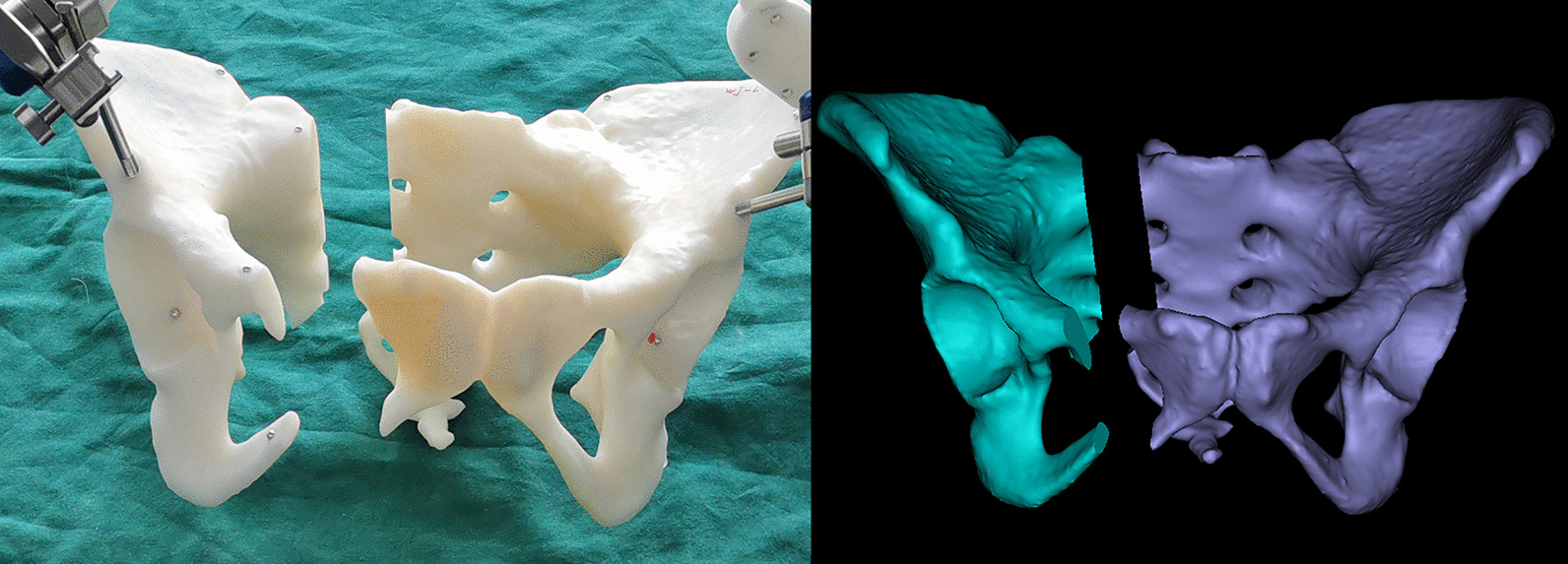
Real-time 3D navigation

An optical tracking marker (Tracker) was installed on both side iliac crest of the pelvic model; the CT data and Cone-beam CT(CBCT) data of the pelvic model were collected. According to the collected CT data of the pelvic model, we reconstructed both the side pelvis model and Tracker model with medical image processing software. According to the collected CBCT data of the pelvic model, we also reconstructed both sides pelvis model with medical image processing software. Then, the navigation registration software registered the reconstructed high-resolution CT model with the CBCT model, and the position of the pelvic model can be tracked in real-time and displayed on the screen, as shown in Fig. 5.

#### Reduction path planning

By utilizing a reference-coordinate system, the computation of special reduction parameters is possible. These describe the displacement and malalignment in the current position and are used for planning manual and semi-automatic reduction paths. For this, both fragments are visualized on a screen, and the fracture is reduced by changing the reduction parameters until the desired target position is reached.

**Fig. 6:**
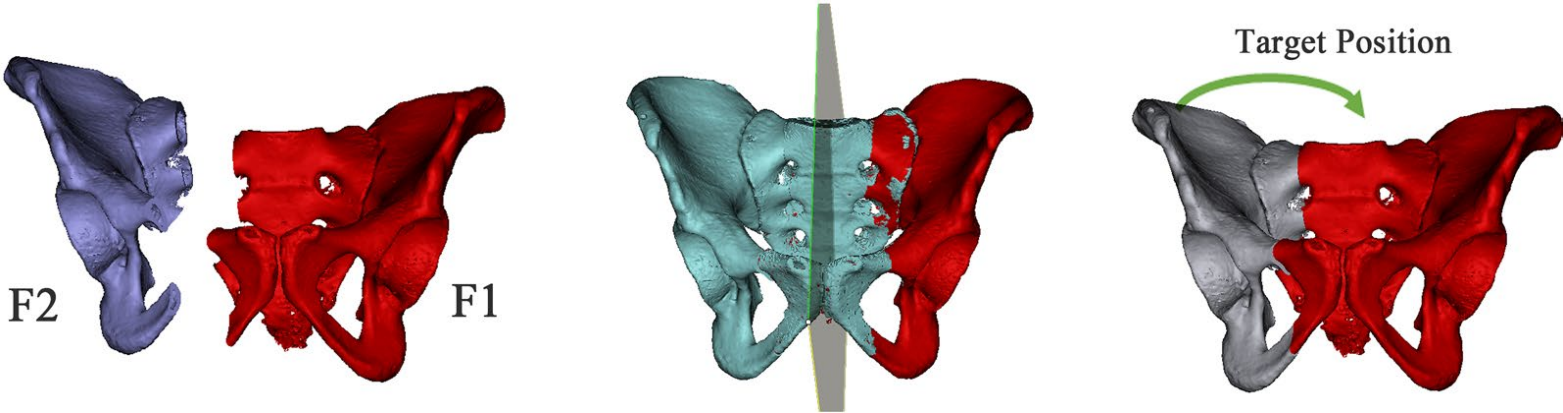
Symmetrical reduction in the pelvis, using physical symmetry of the other side of the pelvis to obtain the reduction target position

Based on the mirror symmetry principle of the human body, the mirror model of the uninjured hemipelvis could be regarded as the anatomical position of the injured hemipelvis [14]. The 3D orientations required to transfer the displaced fracture fragment to its anatomical position can be calculated with the whole-surface registration. The doctor could make a slight adjustment to the target position of F2 and finally generate a clinically satisfactory reduction target position (Fig. 6). Based on the target position, the RAFR system developed an automatic reduction algorithm in which the shortest path planning is adopted, and the planned reduction process was displayed. If any impingement occurs during the reduction process, the surgeon can manually set one or more path points to avoid impingement. The fracture pieces were moved to the path points in sequence and finally reached the target position.

#### Control and fixation

**Fig. 7:**
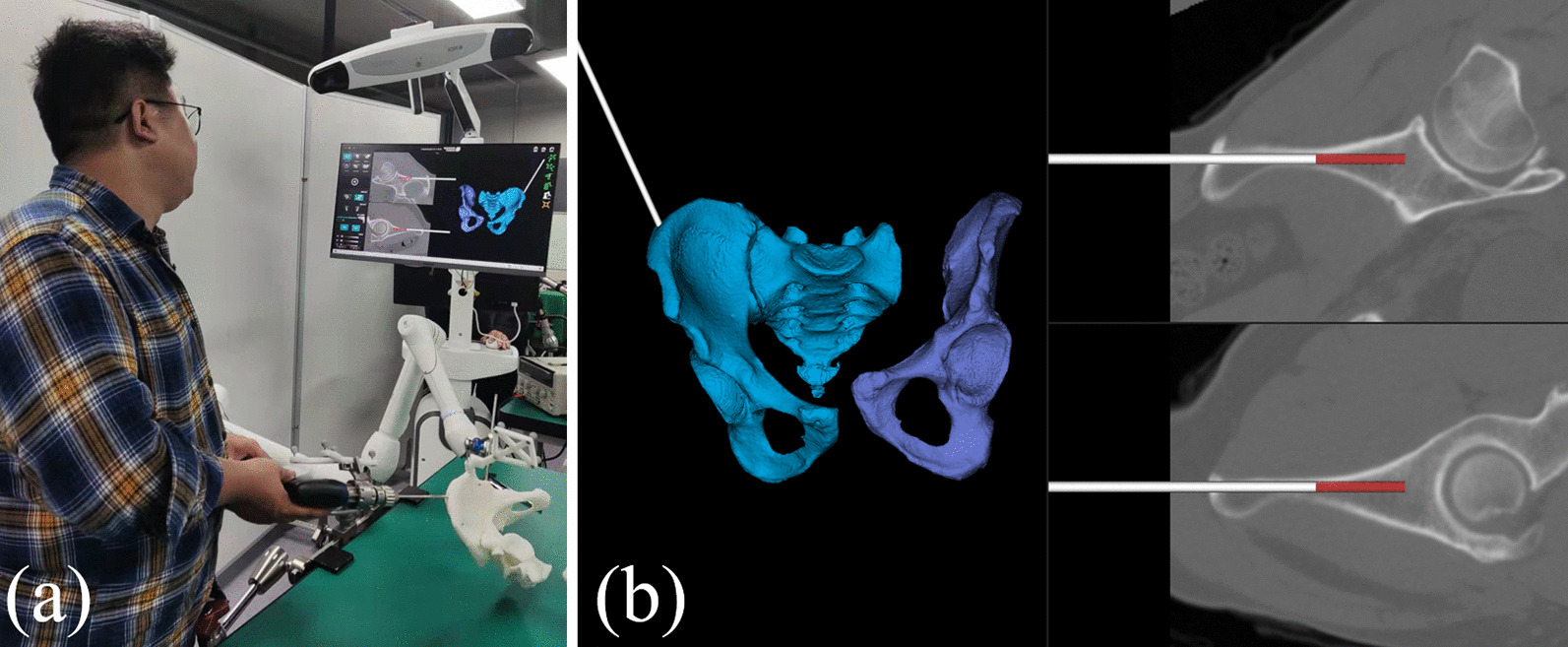
Hand drill navigation system **a** the Schantz pin placement, **b** Real-time display of the guide pin

To achieve the pelvic fracture reduction, we need to maintain F1 fixed and control the F2 fracture fragments to move to the reduction target position. To achieve rapid intraoperative fixation of the healthy side of the pelvis (F1), a 6-DOF quick-holding arm is designed to quickly lock and unlock and provide a holding force of more than 40 kg, replacing the traditional bedside fixed frame. Schantz pins were usually used clinically to control the fracture fragments. In this research, we have designed a hand drill navigation system. The navigation software could calculate the optimal position of Schantz pins placement. A tracker was installed on the hand drill, and then, the software can display the position and depth of the Schantz pin in real-time, as shown in Fig. 7.

#### Robotic-assisted fracture reduction

**Fig. 8:**
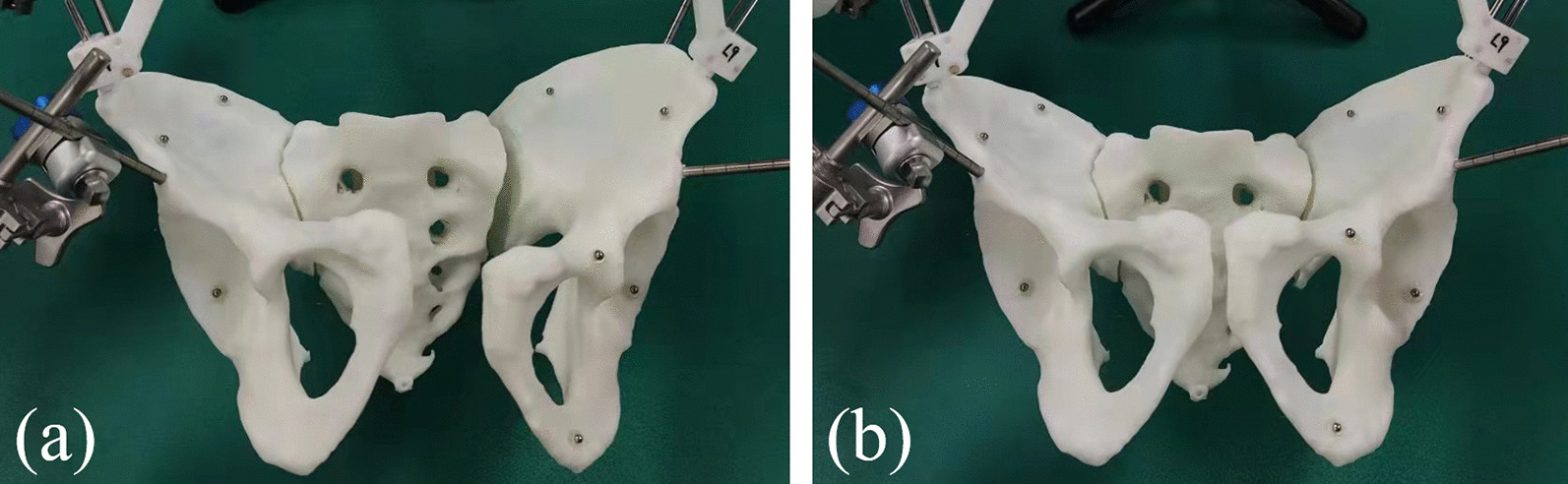
The fracture reduction was completed by robot system. F1 and F2 were controlled by holding grip end-effector and robot, respectively. **a** Simulation of the displacement of a real pelvic fracture. **b** Complete fracture reduction by robot system

Two Schantz pins were inserted into F1 and F2, respectively, to control the pelvis during the operation. The two Schantz pins on F1 were locked with the quick grip end-effector on the holding system to fix the healthy side during the reduction process. The two Schantz pins on F2 were connected with the end-effector on the robot to control the movement of F2. The optical tracking tool is installed on the robot's end to track the end-effector reduction movement. After the F1 holding and fixing, the robot moves along the planned path, and the software displays the moving position of the pelvis in real-time. Finally, the fracture fragments moved to the target position, and the robot's active reduction was completed, as shown in Fig. 8.

## Experimental evaluation

### The accuracy study of navigation

The navigation accuracy was an essential criterion for evaluating the performance of the reduction robot system. The accuracy of a navigation system was usually assessed by the registration accuracy between the preoperative or intraoperative image and the real-time position of the targeted bones retrieved by the trackers and intra-operative fluoroscopy. To evaluate the accuracy of the three-dimensional real-time navigation algorithm during the operation, we design the navigation accuracy study. The metal balls and optical tracking marker were installed on the pelvis model, and then, the spiral CT scan was performed for the pelvis model. We measured the relative position relationship between the ball and the optical tracking mark according to the CT data. We then marked the metal ball position as C1, which is regarded as the ground truth of the position before registration. Then, we performed a CBCT scan on the pelvis model and tracker. We registered the CBCT models of pelvis and tracker with CT models of pelvis and tracker, respectively, to obtain the relative position relationship between the tracker and the pelvis, so we completed the intraoperative real-time three-dimensional navigation. The navigation software calculated the coordinates (C2) of the metal balls on the pelvis. The difference between C1 and C2 is the registration error value E1(E1 =|C1–C2|). Each group of models was tested three times, and the average value was taken. To comprehensively analyze the registration error value, we also analyzed and showed the error distribution of the global 3D point cloud error for a model’s registration results.

### The accuracy study of simulated fracture reduction

Since F1 is completely fixed during the operation, we only tested the F2 location in the experiment. Like the navigation accuracy study, we tested and analyzed the system error of the anatomical landmarks using the pre-installed metal balls. The coordinates of the metal balls on F2 were measured before fracture, as the ground truth before reduction (C3). We use the robot system to complete the fracture reduction according to the automatic reduction algorithm. The center coordinates of the metal ball on F2 were measured as the position's value after reduction (C4). The distance between C3 and C4 is the reduction error value E2(E2 =|C3–C4|). Each group of models was tested three times, and the average value was taken. We also analyzed and showed the error distribution of the global 3D point cloud error for a model’s reduction results.

### Statistical analysis

Statistical analysis by analysis of variance was performed using SPSS 25 (SPSS Inc., Chicago, Ill). Data were presented as means ± SD. The error distribution of the global 3D point cloud error was calculated by Geomagic Studio 2013 (Geomagic Inc, USA).

## Results

**Fig. 9:**
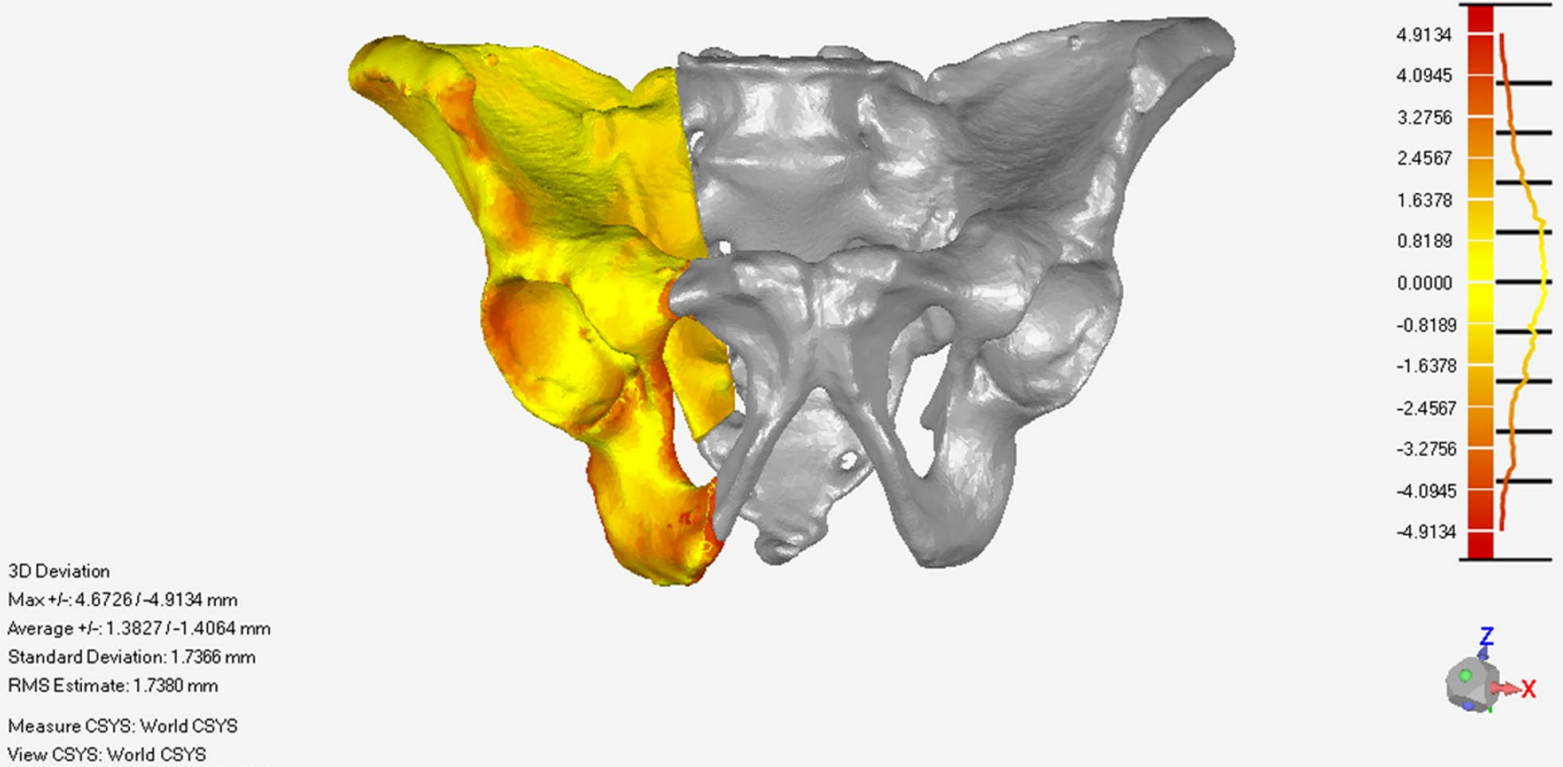
The registration error distribution of the global 3D point cloud on the fracture side of one model

**Fig. 10:**
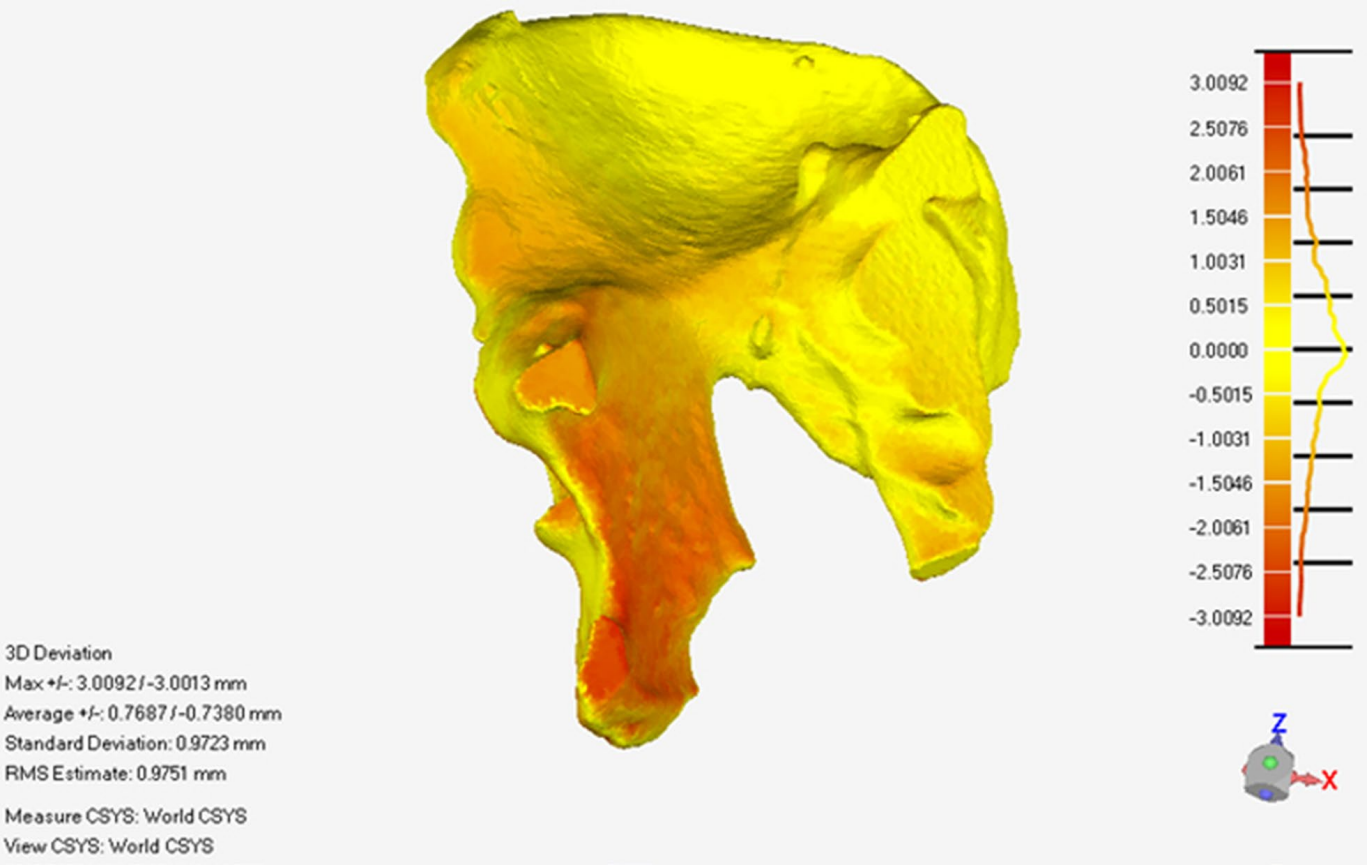
The reduction error distribution of the global 3D point cloud on the fracture side of one model

The mean registration error E1 of the 20 models was 1.29 ± 0.57 mm. The mean reduction error E2 of the 20 models was 2.72 ± 0.82 mm. The mean registration error E1 and reduction error E2 in different anatomy landmark points are shown in Table 1. The registration error distribution of the global 3D point cloud on the fracture side of one model showed as the error value increases, the point cloud color will change from yellow to red(Fig. 9). The reduction error distribution of the global 3D point cloud on the fracture side of one model showed as the error value increases, the point cloud color will change from yellow to red (Fig. 10).

**Table 1:** The mean registration error E1 and reduction error E2 in different anatomy landmark points

System error (mm)	A	B	C	D	E	F	G	H	I
Registration error value E1	0.76 ± 0.24	1.27 ± 0.3	1.1 ± 0.42	0.89 ± 0.21	1.09 ± 0.4	1.45 ± 0.3	0.94 ± 0.27	1.79 ± 0.39	2.29 ± 0.48
Reduction error value E2	2.94 ± 0.77	2.8 ± 0.58	2.3 ± 0.72	2 ± 0.52	2.82 ± 0.73	3.47 ± 0.78			

A, Anterior iliac wing on F1; B Posterior iliac wing on F1; C acetabulum on F1; D sacrum on F1; E superior ramus of pubis on F1; F, inferior ramus of pubis on F1; G, Anterior iliac wing on F2; H, Posterior iliac wing on F2; I, Acetabulum on F2

## Discussion

As we seek to improve further the quality and outcomes of the operations we provide, robotics and artificial intelligence applied to orthopedics have become an exciting topic both from the engineering point of view and the surgical one. The main goal of those systems includes many aspects, such as restoring the limb alignment and physiological kinematics of the joint, providing the robotic support to precisely prepare the bone, pathologic feature recognition of fracture, 3D templating and operative planning, automated image processing capacity to the diagnosis of periprosthetic joint infection, postoperative monitoring and outcome assessment [15–17]. Various robot-assisted orthopedic surgery (RAOS) is currently available on the market, each addressing specific surgeries and characterized by a series of special features that may involve different requirements or modus operandi. RAOS has been utilized in a variety of orthopedic operations, including fracture fixation for traumatic surgery, total hip and total knee arthroplasty [18, 19], spine surgery [20, 21], bone tumor surgery [22], arthroscopy [23], and fracture fixation for traumatic surgery [24]. However, robotic in fracture reduction remains at an infant stage.

Reduction is a crucial step in the surgical treatment of bone fractures to achieve anatomical alignment and facilitate healing. Stable and safe fixation is achieved only after an accurate reduction. Reduction is also considered technically challenging. For pelvic fractures, closed reduction operation is more challenging because most pelvic fractures have ﻿ deep injury location, complex injury mechanism, diversified fracture displacement directions, high reduction resistance [25–28]. A significant difficulty is to deduce the desired target position from medical imaging. This can lead to postoperative malalignment, which significantly impacts the healing course [29, 30]. During fracture reduction procedures, obstacles may be present. High forces caused by excessive stretching of surrounding soft tissues increase the physical load to the surgeons and can prevent the desired movement. An additional obstacle occurs when there are possible fragment collisions. Then, the movement is obstructed, and it is impossible to achieve the desired position. Finally, when an obstacle occurs, a different or an additional action has to be performed. This may lead to a trial-and-error procedure that usually results in increased radiation exposure and a prolonged surgical time.

To mitigate the above disadvantages, researchers developed some reduction robots. However, most current domestic and foreign reduction robots mainly focus on lower extremity diaphyseal fracture. And most of the existing fracture reduction and navigation systems belong to the laboratory and cannot solve the clinical problem of pelvic fracture. Some researchers designed a series of reduction frames that connected the operating table to pelvic external fixators to provide better support for pelvic fracture reduction manipulations.

Matta et al. [31] developed a hip fixation frame that secures the intact hemipelvis to the operating table. With the table-skeletal fixation, the fractured and displaced fragments can then be manipulated around the securely fixed uninjured hemipelvis. Lefaivre et al. [32]upgraded this type of frame to design the Starr frame, which could provide stabilization of the intact hemipelvis to the operating table and facilitate multiplanar reduction in the injured hemipelvis with the use and manipulation of external fixator pins. Tang et al. [33] developed a pelvic reduction frame with one more degree of freedom than the Starr frame. However, limited by the bulky size of the frame, it is challenging to complete the flexible reduction in the multi-dimensional displaced pelvic fracture fragments. With these pelvic reduction frames, surgeons still required complete reduction manually under intraoperative fluoroscopy. The reduction mainly depends on the surgeon’s experience. Therefore, there are certain limitations for the frame in pelvic fractures.

To the best of our knowledge, no 3D-based image-guided intelligent serial RAFR system for pelvic fractures has been reported in the relevant literature. Our intelligent RAFR system was the first 3D-based image-guided serial robot for pelvic fracture reduction. To explore the feasibility of our robot system, we selected 20 cases of healthy pelvis CT data for 3D printing to obtain 20 pelvic models; the pelvic models were osteotomized based on the 3D images of 20 actual pelvic fractures to make the pelvic fracture models for reduction. With the osteotomized pelvic fracture model, it is very convenient for us to evaluate the result of the reduction. The fractured end is very neat; we could easily look directly at the robot's reduction position. So, we can obtain accurate reduction data and compare them with the reduction path planning.

Projecting the 3D pelvic geometry onto a 2D X-ray may not reflect the accurate position of the pelvis, particularly the complex posterior rings. The comprehensive and complicated pathologies associated with unstable pelvic fractures require a 3D-based registration method for precision and accuracy. The registration is a crucial part of the clinical workflow, which considerably affects the pelvic fracture reduction accuracy. Accurate image registration can enable us to accurately judge the displacement of fracture fragments through navigation image, the location of holding pin placement, the real movement path of displaced fracture fragment during reduction, and the final reduction result.

Our study proposed a new registration method based on preoperative CT and intraoperative CBCT. We could establish the relative positions of the pelvis and optical markers to realize real-time tracking of the pelvis. We also made a particular verification procedure for the accuracy of image registration. The following reduction procedure could only be carried out when the registration accuracy meets the surgical requirements. In our study, the registration was successfully executed on all the models, showing that it is reliable and can achieve intra-operative registration with a high level of accuracy, providing a registration error of only 1.29 ± 0.57 mm. Han et al. reported a method to automatically compute the target pose of dislocated bones in preoperative CT and provide 3D guidance of reduction using routine 2D fluoroscopy. Their phantom study demonstrated a mean registration error of 2.5 mm. Our registration method was more accurate than Han’s. Moreover, our new registration method was more in line with clinical norms and more likely used in the clinic. Compared with the technique of implanting optical or metal markers on the bone before CT scan [34, 35], the registration of our system did not require patients to implant markers before surgery which reduces the patient's pain and the risk of infection.

﻿Reduction is a crucial step in fracture treatment and is considered to be demanding. Various computer and robot-assisted approaches were developed to avoid reduction-related problems [36–39]. Despite all advances in the reduction-related field, problems that may occur during the actual reduction procedure are studied insufficiently. The resistance to reduction comes primarily from the muscle force. If the reduction path is not planned reasonably, the resistance could be sufficiently large to injure the soft tissue, especially the nervous tissue, causing irreversible nerve and tissue damage. Therefore, the definition of reduction paths is regarded as a critical methodology for analyzing reduction-related problems and further improving reduction procedures [40].

Unlike traditional closed reduction surgery and frame-assisted closed reduction, which relied on the surgeon’s experience and repeated fluoroscopy. The advantages of our system are intelligentization and personalization. The mirror-symmetric theory of bilateral lower extremities has been proposed by previous researchers and used to direct the navigated or robotic fracture reduction [41–45]. During reduction path planning, the mirror model of the intact bone was considered to be the reference of the fractured bone for navigation. The target position of reduction is computed first. By knowing the desired target position, the shortest optimal paths of fracture reduction are analyzed and planned to reduce the fracture fragments accurately. At the same time, the surgeon can design one or more waypoints to avoid bony impact. The planned paths can be used for navigated fracture reduction based on their experience. By semi-automatic movement patterns, the fracture can be reduced step by step in a simple way. For this, fragments are visualized on a screen, and the fracture is reduced until the exact target position is reached. Unnecessary and prohibited movements can be avoided.

To facilitate the reduction in the pelvis by the mechanical arm, we designed a special holding system to control the healthy hemipelvis. The holding system can be easily and rigidly fixed to the operating table. At the same time, the holding arm has 6 DOF, which could lock rigidly to the holding pin on the pelvis easily. So we could have stable and robust holding and fixation for the pelvis.

The soft tissue connected to the fracture fragments creates reactive power to the reduction force, which is applied to the fragment through the manipulating pin. If the Schantz pin is not connected to the robot end-effector or the fragment rigidly, the reduction force will not translate to the fragment, potentially compromising the reduction procedure. So optimal trajectory of the manipulating Schantz pin placement was critical. Our trajectory of the manipulating pin placement is designed according to the three-dimensional CT image before the operation. After registration with the intraoperative image, our system can provide an intraoperative navigation trajectory of the manipulating pin for the stable holding of the pelvis. The optimal trajectory of the manipulating pin placement is conducive to the maximum utilization of the robot's reduction force. At the same time, it reduces the loosening of the holding pin or iatrogenic fractures due to the poor position of the pin.

The reduction manipulator arm of the robot is the UR manipulator arm commonly used in medical robots [46]. We designed a special gripping end-effector at the end of the manipulator's arm. The 2–3 adjustable robot connecting rods on the gripping end-effector are connected with the 2–3 holding pins on the displaced pelvis, respectively, and stably. After the connecting rods and holding, pins are locked, and the reduction manipulator arm and the displaced pelvis can be firmly connected. As the reduction manipulator arm moves according to the planned path, the displaced pelvic part can move according to the reduction path.

The metric chosen for the reduction error accuracy evaluation shows how far the manipulated fragments are from the desired, reduced position. Grading systems for evaluating the pelvic fracture reduction quality have been proposed by Matta et al. [47, 48], Majeed et al. [49], Lindahl et al. [50], and Tornetta et al. [9]. According to the Matta Criteria, the most used grading system, reductions were graded by the maximal displacement. The criteria were: excellent 4 mm or less, good 5 to 10 mm, fair 10 to 20 mm, and poor more than 20 mm. Given the abovementioned criteria, the mean reduction error of 2.72 ± 0.82 mm found in our study could be considered that clinically excellent reduction was achieved in all 20 models.

The global error analysis of registration and reduction results showed that as the error value increases, the point cloud color will change from yellow to red. As shown in Figs. 9 and 10, the red color which means higher errors is mainly located at the edge of the pelvis, such as the iliac wing. These may be caused by the mirror symmetry principle. The overall pelvis is symmetrical, but the details are different. There are more details on the edges. Therefore, when the reduction planning algorithm was based on the mirror symmetry principle, some minor errors in the calculation results must be made. However, these minor errors were usually within a clinically acceptable range. In the future, statistical methods will improve the reduction target planning algorithm to improve accuracy.

Compared with the other robot system, our reduction system also had the advantage of saving space. Especially after the fracture reduction is completed, there is enough space left so that the doctor can smoothly complete the minimally invasive fixation of the fracture and achieve the ultimate treatment goal.

### Limitations

In our study, the pelvic fracture model was very light. No soft tissue connected to the fracture fragments could not create reactive forces to the reduction force. Without the reactive forces of soft tissue, the maximum reduction force in our robot reduction system is less than 10N. During the actual reduction in a pelvic fracture, one of the difficulties is the high forces that occur during fracture reduction. These high forces increase the physical load of the surgeons and can prevent reduction movements [51]. Therefore, we must consider this situation in actual clinical practice. However, too much higher mechanical loads will also increase the risk of injury to patients, and safety is the most critical issue for a medical device. At present, the maximum robotic load of our reduction system is 160N which is higher than the latest version of the serial robotic arm by Oszwald et al. [37]. The serial robotic arm developed by Oszwald can output a maximum load of 110N. In our actual clinical operation, we also use the active lower limb traction to help reduce, it is expected that our robotic system can meet the requirements of clinical operations. With further clinical trials, our team will obtain relevant data on the reduction force and optimize our robot system based on clinical practice to fulfill better the clinical needs of minimally invasive reduction in pelvic fractures. Our robotic system could decrease the cumulated radiation dose for medical staff. However, intra-operative CT scan was needed during the operation, which brought more radiation to the patients. Considering that the intraoperative CT scan brings accurate registration and reduction, which will bring good clinical outcomes to the patient, it is worthwhile to perform the CT scan during the operation. Meanwhile, although no intra- or inter-observer reliability studies of our reduction system have been performed, the system itself will not cause any intra- or inter-observer differences. Suero et al. [52] demonstrated that this kind of robot was reliable and reproducible enough, and extensive training was not required before the application. We also have designed and combined all of the necessary functions of the abovementioned systems into a self-developed program to simplify the use of this system. Therefore, the performance results of the surgeons who use this reduction system often should be the same as those of untrained surgeons.

## Conclusion

This study presented a self-developed robot reduction system for pelvic fracture. We realized intraoperative real-time 3D image navigation. With our robot reduction system, the computation of particular reduction parameters and thus, the intelligently planning of reduction paths was made, which allows analyzing and planning the tactical procedure of a fracture reduction preoperatively. We designed a special holding system to control the healthy hemipelvis and a reduction manipulator arm to reduce the fracture fragments. Then, we successfully reduced the fracture fragment with our robot reduction system according to the planned path. The accuracy of image registration error and fracture reduction error in our study was excellent, which could reach the requirements of the clinical environment. In summary, our study demonstrated the accuracy and effectiveness of our robotic reduction system and its applicability and usability in clinical practice, thus paving a way toward robot minimally invasive pelvic fracture surgeries.

## Data Availability

The datasets generated and analyzed during the current study are available from the corresponding author on reasonable request.

## Abbreviations

DOF: Degree-of-freedom
CBCT: Cone-beam CT
DICOM: Digital imaging and communication in medicine
3D: Three-dimensional
RAFR: Robot-assisted fracture reduction
